# Health Professionals’ Views on the Use of Conversational Agents for Health Care: Qualitative Descriptive Study

**DOI:** 10.2196/49387

**Published:** 2024-09-25

**Authors:** A Luke MacNeill, Lillian MacNeill, Alison Luke, Shelley Doucet

**Affiliations:** 1 Centre for Research in Integrated Care University of New Brunswick Saint John, NB Canada; 2 Department of Nursing and Health Sciences University of New Brunswick Saint John, NB Canada

**Keywords:** conversational agents, chatbots, health care, health professionals, health personnel, qualitative, interview

## Abstract

**Background:**

In recent years, there has been an increase in the use of conversational agents for health promotion and service delivery. To date, health professionals’ views on the use of this technology have received limited attention in the literature.

**Objective:**

The purpose of this study was to gain a better understanding of how health professionals view the use of conversational agents for health care.

**Methods:**

Physicians, nurses, and regulated mental health professionals were recruited using various web-based methods. Participants were interviewed individually using the Zoom (Zoom Video Communications, Inc) videoconferencing platform. Interview questions focused on the potential benefits and risks of using conversational agents for health care, as well as the best way to integrate conversational agents into the health care system. Interviews were transcribed verbatim and uploaded to NVivo (version 12; QSR International, Inc) for thematic analysis.

**Results:**

A total of 24 health professionals participated in the study (19 women, 5 men; mean age 42.75, SD 10.71 years). Participants said that the use of conversational agents for health care could have certain benefits, such as greater access to care for patients or clients and workload support for health professionals. They also discussed potential drawbacks, such as an added burden on health professionals (eg, program familiarization) and the limited capabilities of these programs. Participants said that conversational agents could be used for routine or basic tasks, such as screening and assessment, providing information and education, and supporting individuals between appointments. They also said that health professionals should have some oversight in terms of the development and implementation of these programs.

**Conclusions:**

The results of this study provide insight into health professionals’ views on the use of conversational agents for health care, particularly in terms of the benefits and drawbacks of these programs and how they should be integrated into the health care system. These collective findings offer useful information and guidance to stakeholders who have an interest in the development and implementation of this technology.

## Introduction

### Background

Global health spending has reached record levels and is expected to increase in the coming years [1,2]. Despite this enormous investment, health care systems around the world are under pressure as they struggle to cope with health worker shortages [3,4], health system overuse [5,6], and a growing burden of noncommunicable diseases [7,8], among other challenges. These challenges and the pressure that they place on health care systems can have consequences for people providing and accessing health services. For instance, health professionals face heavy workloads and high levels of exhaustion and burnout [9-12]. In addition, individuals who are seeking care often experience long wait times that can negatively impact their health and well-being [13,14].

To reduce strain on health services and ensure the safe and efficient delivery of care, health care systems need to find accessible and cost-effective ways to supplement and support traditional forms of health care. Conversational agents are an emerging technology that might help. Broadly speaking, conversational agents are automated software programs that are designed to engage in humanlike conversation with people [15,16]. Common examples include text-based chatbots [17], voice-activated virtual assistants [18], and “embodied” conversational agents that appear in virtual or animated bodies [19,20]. Whereas standard computer interfaces support a somewhat rudimentary and impersonal interaction with users, conversational agents provide users with the impression of interacting with a real human. This quality would presumably make these programs well suited for dealing with sensitive issues or topics, such as those that arise within a health context.

In recent years, conversational agents that are used for health care have become more common in clinical and commercial settings [16,21-23]. Although the literature on these programs is still growing, current evidence suggests that they are an effective tool for health promotion and service delivery across a range of health areas [24-26]. For instance, they have been used to promote physical activity [27,28] and weight loss [29,30], support chronic disease management [31,32], provide preconception care [33,34], and improve mental health [35,36]. Research also shows that people who use these programs tend to have positive perceptions and opinions about them, as well as a desire or intention to continue using them in the future [16,37,38].

One aspect of conversational agents that tends to get overlooked in the literature is the views of health professionals toward these programs. Health professionals have considerable experience with the management and delivery of health care and may be able to offer unique insight into the use of this technology for health purposes. To date, only a handful of studies have considered health professionals’ views in this area [39-41]. These studies had a narrow scope in terms of the specific types of health professionals that were assessed (eg, mental health professionals). In addition, much of this research captured participants’ views using structured questionnaires with predefined questions and response scales [40,41]. These questionnaires allowed for standardized and easily aggregated data, but they may not have covered topics or points that would have emerged organically through other means, such as qualitative interviews. Due to these limitations, there is currently a fragmented and incomplete understanding of health professionals’ views surrounding this technology.

### Objectives

The purpose of this study was to gain a more comprehensive understanding of health professionals’ views on the use of conversational agents for health care. We recruited health professionals with a variety of professional backgrounds and asked them to share their views via semistructured open-ended interviews. We were interested in their views on 2 specific subjects. First, we wanted to learn more about the potential benefits and drawbacks of using conversational agents for health care. Second, we wanted to gain a better understanding of how to best integrate conversational agents into the health care system. Capturing health professionals’ views on these subjects was expected to provide useful insight into whether and how these programs should be implemented in practical settings.

## Methods

### Study Design

We used a qualitative descriptive design to explore health professionals’ views on the use of conversational agents for health care. The goal of qualitative description is to provide a rich, detailed description of a phenomenon, emphasizing surface versus more interpretive readings of the available data in an effort to preserve participants’ voices and perspectives [42,43]. It is viewed as a useful approach for answering questions relevant to practitioners and policy makers [42] and therefore well suited for this study. All data for this study were collected using cross-sectional semistructured interviews (details in the Procedure section). The study is reported in accordance with Standards for Reporting Qualitative Research guidelines [44].

### Participants and Recruitment

We recruited Canadian health professionals for the study, specifically physicians, nurses, and regulated mental health professionals (eg, clinical psychologists). No prior experience with conversational agents was necessary for participation. Participants were recruited through social media posts, classified websites, e-newsletters, and emails to relevant organizations. The initial recruitment target was 24 participants, divided evenly between physicians, nurses, and regulated mental health professionals. Once these participants were recruited and interviewed, we evaluated the interview data to determine whether additional participants should be recruited. We decided that no further participants were required, as no new themes were identified in the last few interviews (ie, thematic saturation was reached) and we felt that the data set was sufficiently rich to address the research objectives.

### Procedure

Prospective participants contacted the research team to learn more about the study and set up a web-based interview. Prior to the interview, participants were emailed a PDF document that provided information about conversational agents and illustrated several conversational agents that are used for health care. The purpose of the PDF document was to familiarize participants with these types of programs before the interview. The document covered conversational agents that support the prevention and treatment of specific health conditions, as well as conversational agents that promote healthy lifestyle habits (physical activity, etc). It included screenshots of both embodied and disembodied conversational agents. Screenshots were drawn from several publications in this area [34,45-50]. In some cases, the images from these publications were replaced with higher-quality versions of the same or similar images that were available from web-based sources (eg, researcher websites).

Participants were interviewed individually using the Zoom (Zoom Video Communications, Inc) videoconferencing platform. Interviews took place between March and September 2021. The same general procedure was used for all interviews. The interviews began with personal introductions and a review of the study information. Next, verbal consent was obtained from participants and the interview questions were administered. Question administration lasted between 10 and 35 minutes (depending on the participant) and followed a semistructured format. Participants were asked about the potential benefits and drawbacks of using conversational agents for health care, as well as the best way to integrate conversational agents into the health care system. See Multimedia Appendix 1 for a full list of the interview questions. At the end of the interviews, participants were given an opportunity to ask any questions that they had.

### Data Analysis

A research assistant (SY) transcribed the interview recordings verbatim, and the transcripts were reviewed by the first author for accuracy. The transcripts were uploaded to NVivo (version 12; QSR International, Inc) for thematic analysis. The thematic analysis followed the 6 phases described by Braun and Clarke [51,52], as follows. The first and second authors familiarized themselves with the data set by reading and rereading the transcripts and making preliminary notes (phase 1). Next, they coded the data set via an iterative process (phase 2). They started by reviewing the transcripts of the first 3 interviews to generate preliminary codes and working definitions. This initial analysis guided the coding of the full data set, which was performed by the first author. The second author independently coded 25% of the transcripts and found a high level of agreement with the first author (Cohen κ=0.81; agreement weighted by source size=99.74%). The 2 coders refined the coding guidelines to address the few disagreements and applied the refined guidelines to the full data set. Next, they worked together to generate initial themes (phase 3); further develop and review the themes (phase 4); and refine, define, and name the themes (phase 5). Finally, the first author wrote the initial draft of the manuscript and all authors performed manuscript revisions (phase 6).

### Trustworthiness

Trustworthiness is a broad concept that refers to the credibility, confirmability, dependability, and transferability of research [53,54]. We sought to establish trustworthiness in our study through the use of several strategies, including investigator triangulation (eg, interrater coding), the creation of an audit trail, purposive sampling of different participant groups, the use of verbatim quotes to support the themes, and peer debriefing with digital health researchers in both the academic and commercial spaces. To enhance methodological rigor, the data analysis was conducted by 2 researchers with different professional backgrounds, specifically the first and second authors. The first author is a health researcher with a focus on digital health research. He has led several studies on the use of conversational agents for health care, including an evaluation study and a scoping review. The second author is a health researcher with a more general focus on access to health services. Her knowledge of digital health and digital health research is more limited. Whereas the second author is primarily a qualitative researcher, the first author is a quantitative researcher with some qualitative experience.

### Ethical Considerations

This study was approved by the research ethics board at the University of New Brunswick (009-2021). The research ethics board provided a delegated review due to the low-risk nature of the study. Informed consent was obtained from all study participants (details in the Procedure section). Transcripts were deidentified during the transcription process to protect participant confidentiality, and interview files were stored on a secure Microsoft SharePoint site. Participants received an entry into a draw for a CAD $100 (US $72.61) Amazon.ca gift card for their participation in the study.

## Results

### Participant Characteristics

A total of 24 health professionals completed the study. Participants included 8 physicians, 8 nurses, and 8 regulated mental health professionals (2 clinical psychologists, 2 psychotherapists, 2 counselors, and 2 clinical social workers). The sample consisted of 19 women and 5 men with a mean age of 42.75 (SD 10.71) years. Participants were from 7 Canadian provinces: Alberta, British Columbia, New Brunswick, Nova Scotia, Ontario, Prince Edward Island, and Saskatchewan. All participants reported that they had used conversational agents in the past, although only 2 had used a conversational agent for health care specifically.

### Benefits of Conversational Agents

We identified 2 themes related to benefits of using conversational agents for health care: *increased access to care* and *workload support for health professionals* (see Table 1). More details on these themes are provided in the following subsections.

**Table 1:** Outline of themes and subthemes grouped by research objective.

Research objective	Theme (subthemes)
Benefits of conversational agents	Increased access to care (convenience and availability, rural access to care) Workload support for health professionals
Drawbacks of conversational agents	Added burden on health professionals Limited capabilities of conversational agents (technological limitations, inadequate care)
Integration into the health care system	Health professional oversight Routine or basic tasks (screening and assessment, information and education, support between appointments)

#### Increased Access to Care

Participants said that conversational agents could benefit patients or clients by providing increased access to care. Most participants who discussed this theme focused on the convenience and availability of these programs, although some participants discussed rural access to care as well.

##### Convenience and Availability

According to participants, conversational agents are able to provide patients or clients with convenient access to care. In other words, people can access these programs whenever they want, regardless of the day or time. For instance, one participant pointed out that conversational agents are available “anytime … 24/7” (Participant 18, nurse), and another participant stated: “To have access to something 24 hours a day is certainly beneficial” (Participant 1, nurse). Some participants contrasted conversational agents with health professionals, many of whom adhere to scheduled office hours. For instance, one participant said that conversational agents “would be always on, not dependent on the hours that health care professionals keep” (Participant 13, physician). By providing convenient access to care, these programs would allow patient or clients to avoid the wait times that are often experienced when seeking an appointment with a care provider: “Wait times are certainly an issue for people, being able to access a provider…. Perhaps these particular types of programs … would improve access [to care] for patients that might have difficulty connecting with a health care provider” (Participant 11, nurse).

##### Rural Access to Care

Participants noted that people who live in rural areas often have more limited access to care than people who live in urban areas, and that conversational agents could be one means to address this disparity. For instance, one participant said, “I think in certain ways it [the use of conversational agents] can be helpful for people if they live in a rural area and they don't have access to a specialist or they don't have a health care provider that they need” (Participant 4, psychotherapist). Other participants reinforced this statement, saying that these programs “might be able to reach more geographical locations that we can’t reach” (Participant 5, clinical social worker), and that they would be useful “if you live way out in the boondocks and can’t get into town” (Participant 21, clinical psychologist). Notably, most of the participants who discussed rural access to care were regulated mental health professionals.

#### Workload Support for Health Professionals

Participants said that conversational agents could provide health professionals with workload support. Although this point was discussed by all 3 health professional groups, it was particularly common among physicians and nurses. Participants noted that health professionals are often overworked and exhausted, and that conversational agents may be able to ease their workload to a certain extent. As one participant said, “It can take some of the pressure off of actual providers, if people are using these chatbots and whatnot. Maybe some of the demand goes down” (Participant 4, psychotherapist). By easing demand for health services, these programs would allow health professionals to devote more time and resources to people who have greater or more urgent needs. For example, one participant said, “[You] may be able to filter some of the more basic health care concerns, if you will, to this type of platform and then allow for health care providers to spend time with patients that have greater needs or need more time in-office or in-clinic” (Participant 11, nurse). One participant illustrated this point by sharing a story about a patient who was seriously injured after failing to get an appointment for a mental health medication refill:

[The patient] said “I call my family doctor, I don’t get ahold of him. My psychiatrist left.” You know, here’s a guy where, maybe if we have AI taking the burden off the family doctor... maybe the guy calls, and the family doctor’s able to see him and this problem never happens.

Participant 3, physician

### Drawbacks of Conversational Agents

We identified 2 themes related to drawbacks of using conversational agents for health care: *added burden on health professionals* and *limited capabilities of conversational agents*. More details on these themes are provided in the following subsections.

#### Added Burden on Health Professionals

Although many participants thought that conversational agents could help ease their existing workload, some were wary that these programs could create new burdens that would strain their time and attention. For instance, participants anticipated that they would be receiving questions about conversational agents from their patients or clients, and that answering these questions would take time out of their workday. As one participant said, she expected “more questions from the patients about it, which ends up taking a lot of time for me” (Participant 13, physician). Participants also recognized that they would need to familiarize themselves with these programs so that they could answer patient or client questions, which would require a further time investment: “People may have questions about it, and if you don't take the time to familiarize with everything, you may not be able to answer them” (Participant 12, physician). Integrating conversational agents into their daily workflow was a concern as well. For instance, one participant worried about keeping on top of patient-related reports that might be supplied by these programs: “It would be an extra layer of my daily work that is time sensitive, you know? Like, every day at three, I have to make sure that I've looked at the last 24 hours of these things” (Participant 23, physician). Finally, some participants were concerned that they would have to spend time and resources addressing health issues that arise from the improper or incorrect use of a conversational agent: “If they [patients or clients] self-selected inappropriately to be seen by a virtual interface, and they delayed care and deteriorated … that would make my job a lot more complex” (Participant 13, physician).

#### Limited Capabilities of Conversational Agents

Participants spent a great deal of time discussing the capabilities of conversational agents. They had many concerns about the technological limitations of these programs, as well as the consequences of these technological limitations, particularly the potential for inadequate care.

##### Technological Limitations

Participants highlighted several technological limitations of conversational agents. Some participants focused on the emotional limitations of these programs, noting that they are unable to experience or convey genuine empathy or compassion: “When we’re at our most vulnerable moments, we expect compassionate care, and there's a limit to what you can get from a … conversational agent” (Participant 19, nurse). Others touched on this same topic from an interpersonal standpoint, commenting on “that loss of human connectedness, that human element” (Participant 11, nurse). Participants also discussed the sensory limitations of conversational agents, particularly their inability to detect important nonverbal cues from patients or clients: “I would wonder about a chatbot’s ability to notice nuance, to hear a voice choking … or to see eyes welling with tears” (Participant 6, clinical social worker). These nonverbal cues include cues that signal deception or dishonesty: “They [patients or clients] say they’re fine, but they actually look like they just dragged themselves out of bed and they haven’t showered in six days … I don’t know how good computers are at picking up that kind of ‘faking it’ behavior” (Participant 21, clinical psychologist). Finally, participants discussed the conversational limitations of these programs, doubting their ability to handle the complex question and answer exchanges that characterize health care interactions: “There’s no actual ability to have context-driven questioning, follow-up, reflection on what the person is hearing” (Participant 8, clinical psychologist).

##### Inadequate Care

Participants were concerned that the technological limitations of conversational agents would result in inadequate care for patients or clients. They were especially worried about patients or clients who are dealing with serious health issues. One participant provided an example within a mental health context:

If someone has really mild anxiety or mild depression, I think something like that could be a really great resource for them…. Whereas on the other hand, someone who is dealing with complex trauma or really significant mental illnesses, there’s no possibility that something automated could provide the care for that.

Participant 4, psychotherapist

A further point of concern was the possibility that patients or clients might not recognize that they are receiving inadequate care, which could compound their health issues. As one participant said, “They feel like what they're getting is enough and it may not actually be enough” (Participant 14, psychotherapist). One participant speculated about a patient’s thought processes in these situations: “‘Robot nurse Becky told me that I’m totally fine. So I’m just not going to go in to the doctor. I’m not going to call telehealth’ … I think it can have the potential of, I guess, false reassurance” (Participant 10, nurse).

### Integration Into the Health Care System

We identified 2 themes related to the integration of conversational agents into the health care system: *health professional oversight* and *routine or basic tasks*. More details on these themes are provided in the following subsections.

#### Health Professional Oversight

Participants said that health professionals should have some oversight of conversational agents that are used for health care. For instance, many participants suggested that health professionals should have input into the development process so that there is some assurance that the program content is up-to-date and correct. As one participant commented, “It needs to have the signoff of a health care professional, somebody that tests it and can ensure all the information is accurate” (Participant 10, nurse). Participants also said that health professionals should have clinical oversight of these programs, which would allow them to evaluate whether a particular conversational agent is suitable for a particular patient or client: “The patient or client should come and see the health care professional and be referred to the program as opposed to just self-referral…. That way, at least someone has evaluated ‘Is this appropriate for that person?’” (Participant 12, physician). Clinical oversight would also allow health professionals to evaluate whether the information supplied by a program is consistent with the information they themselves provide to a patient or client. For example, one participant stated, “I would want to see what kind of information they [patients or clients] were getting, and whether it fit with the way I saw things” (Participant 21, clinical psychologist).

#### Routine or Basic Tasks

Participants said that conversational agents would be most appropriate for conducting routine or basic tasks. In the words of participants, these tasks would include “tedious jobs” (Participant 15, nurse), “basic things” (Participant 19, nurse), and “routine care” (Participant 13, physician). Although participants mentioned several tasks that would be appropriate for conversational agents, their reports tended to center on 3 general areas: screening and assessment, information and education, and support between appointments.

##### Screening and Assessment

Participants said that conversational agents could be used for screening and assessment purposes. For instance, these programs could screen patients or clients who have reached a specific service to see if the service is aligned with their needs: “I see it as a front-end thing where … you’re doing some screening, like, ‘Is this person in the right place?’” (Participant 21, clinical psychologist). In a similar manner, conversational agents would be able to screen individuals who are trying to access a particular program to see whether they meet the program qualifications: “In the program that I work with, we … really have to scope it down to those clients who qualify. So I think it might help us a little bit with filtering clients” (Participant 7, nurse). Once patients or clients have reached an appropriate program or service, conversational agents could perform an initial assessment to determine the severity of their health issues and the urgency of their needs. As one participant said, “It’s like that initial step to gather information and assess sort of the immediacy of response” (Participant 23, physician).

##### Information and Education

Participants said that conversational agents could be used to provide basic health information and education to patients or clients. For example, one participant said, “I can see the role it can play in basic patient education” (Participant 19, nurse), and another participant stated, “I think if people were seeking general health information, then that might be certainly appropriate” (Participant 11, nurse). Several participants provided examples of health areas where conversational agents could be applied. Many of these participants thought that these programs could be used to educate people on the prevention and management of chronic diseases, particularly diabetes, and to provide information to patients prior to and after surgery. One participant touched on both of these points: “In terms of basic health care stuff like diabetic education, pre- and post-op teaching … I think that it would be okay there” (Participant 1, nurse). Participants also thought that conversational agents could be used for psychoeducation and for educating people on health promotion topics such as physical activity and smoking cessation.

##### Support Between Appointments

Participants said that conversational agents could be used to support patients or clients between appointments. This point was especially common among regulated mental health professionals, who thought that these programs would be useful for “between-session maintenance” (Participant 4, psychotherapist) and “outside-of-session work” (Participant 14, psychotherapist). One participant provided a detailed example of how they might be used:

They’d be great for homework things, I think. So often, you check in with people and they’re like, “Well, I kind of forgot what you told me” or “Oh, I didn’t do it” … Having something that would have reminders, different pieces of homework that would pop up, I think that would be great.

Participant 9, counselor

In addition to addressing the topic of between-session work, this participant’s quote highlights the importance of patient or client accountability. Participants thought that conversational agents could be used to remind individuals about any necessary work to be done between appointments with a care provider. For instance, one physician said, “I think that would be helpful, as like that little at-home reminder of ‘This is what I was supposed to do for my exercise,’ or whatever …. Something that keeps them accountable at home, once they leave my office” (Participant 24, physician).

## Discussion

### Principal Findings

The results of this study provide insight into health professionals’ views on the use of conversational agents for health care, particularly in terms of the benefits and drawbacks of these programs and how they should be integrated into the health care system. Participants said that conversational agents would be useful for increasing access to care for the public, including rural populations that often face access issues. They also said that these programs could provide health professionals with some much-needed workload support. However, participants were wary of any added burden on health professionals, such as the need for program familiarization and having to integrate this technology into their workflow. They were also mindful of the technological limitations of these programs and the implications of these limitations for patients or clients. To help safeguard patient or client welfare, participants said that health professionals should have some oversight in terms of the development and implementation of these programs. They also suggested that the use of conversational agents should be limited to routine or basic health care tasks, such as screening and assessment, providing information and education, and supporting individuals between appointments.

### Comparison With Prior Work

The current results reinforce many findings from past studies in this area, despite important differences in the scope and methodologies of these studies. For instance, similar insights were reported by Palanica et al [40], who administered a web-based survey to general practitioners; Sweeney et al [41], who distributed a web-based survey to mental health professionals; and Barnett et al [39], who conducted in-person focus groups with substance use counselors. Across these studies, participants said that conversational agents could benefit patients or clients by improving access to care. They also touched on the limited capabilities of these programs, particularly their inability to understand or convey genuine human emotions. With respect to implementation, the participants in these studies suggested that conversational agents would be best suited for routine tasks, such as providing information or education and supporting people between appointments (eg, sending reminders). All of these topics were major discussion points among the participants in our study.

There were some differences between the current results and past findings as well. Participants in our study emphasized several points that received little attention in previous studies, such as the impact of conversational agents on health professionals’ workloads and the importance of health professional oversight of this technology. At the same time, our participants failed to mention certain other points that emerged in past studies, particularly the studies by Palanica et al [40] and Sweeney et al [41]. For instance, participants in these latter studies expressed concern that patients or clients could abuse conversational agents by self-diagnosing too often, and that they might not feel connected to their actual health care providers if they use these programs. Neither of these points were raised by participants in our study. These divergent findings are likely due to methodological factors. Both Palanica et al [40] and Sweeney et al [41] assessed health professionals’ views using structured surveys with predefined questions and response scales. These surveys may have prompted participants to consider topics that they otherwise might not have thought about, while also limiting the scope and substance of their responses. By comparison, our study used semistructured interviews with open-ended questions, which captured participants’ thoughts more organically.

### Differences Among Health Professionals

Although the results of this study were fairly consistent across physicians, nurses, and regulated mental health professionals, there were some differences between these participant groups. For instance, mental health professionals were more likely than other participants to discuss how conversational agents could improve rural access to care, an outcome that might reflect challenges in delivering conventional mental health services to rural populations [55-57]. Mental health professionals were also more likely than others to say that conversational agents have the potential to support patients or clients between appointments. Most of their comments focused on the topic of between-session work, which speaks to the importance of this work in mental health treatment [58-60]. Meanwhile, discussion of workload support for health professionals was more common among physicians and nurses than regulated mental health professionals. This outcome may reflect the considerable occupational demands of physicians and nurses and the importance of managing time pressures within these professions [61-63]. Taken together, these collective differences suggest that there are profession-specific uses and benefits with respect to conversational agents, in addition to the more general uses and benefits that apply across professions.

### Practical Implications

The results of this study offer insight to stakeholders who have an interest in the adoption and use of conversational agents for health care. For instance, policy makers, institutional leaders, and health professionals will be able to draw on these results when weighing the potential benefits and drawbacks of adopting this technology in practice. These individuals can also look to our results for guidance on how to safely implement this technology; namely, by employing programs that perform routine or basic tasks and by ensuring that these programs have appropriate oversight. Researchers and companies that develop conversational agents for health care might find these results useful as well. Based on our findings, developers should include health professionals in the development process and focus their efforts on programs that pose minimal safety risk to users. They should also clearly communicate the limitations of these programs to users to help prevent instances of inadequate care. By promoting the safe and responsible implementation of conversational agents for health care, developers and other stakeholders can increase the adoption and use of this technology, which will ultimately benefit those individuals who are either accessing or providing care.

### Limitations and Future Directions

Participants in this study worked within the Canadian health care system, and so their views on the benefits and drawbacks of using conversational agents for health care, as well as the best way to implement this technology in practice, were shaped by their experiences within this particular setting. It is unclear whether these results are transferable to people working in other health care systems. It should be noted that many of the current results are consistent with findings from previous studies involving health professionals from other countries [39-41], although these studies had a narrower scope and may not be indicative of broader views on conversational agents within these health care systems. In any case, researchers may want to replicate this study with health professionals in other countries to see whether their views differ.

Another limitation with this study is the fact that many participants had never used a conversational agent for health care. The views shared by these participants could reflect their inexperience and may lack a certain degree of depth or insight. To their credit, all of these participants did have experience with conversational agents in other areas of their lives (eg, customer service chatbots), and so they likely had a reasonable understanding of how this technology would function and perform in a health context. In addition, study participants were shown several examples of conversational agents that provide health care before their interviews to ensure that they had knowledge of these programs and their capabilities. Regardless, future studies should focus on health professionals who have more direct experience with conversational agents in this domain so that there is some assurance that their understanding of these programs is accurate and comprehensive.

A further limitation with this study concerns the demographics of the sample. Although the participants in this study covered a wide range of ages and professions, most of the sample (19/24, 79%) identified as women. This gender imbalance is not particularly surprising, as women occupy the majority of health care positions in Canada [64,65]. However, a sample with a greater number of men may have produced additional or alternate insights. In the future, researchers in this area may want to emphasize men in their recruitment strategies so that they achieve a more gender-balanced sample of participants.

### Conclusions

The results of this study provide insight into how health professionals view the use of conversational agents for health care. The health professionals in this study saw distinct benefits and drawbacks to the use of conversational agents for health care, and they shared several suggestions for safely integrating these programs into the health care system. This collective feedback provides a useful indicator of how conversational agents will be received in health settings in the coming years, and it also offers some guidance on how these programs should be implemented to provide the most benefit to people who are either accessing or providing care.

## Appendix

Multimedia Appendix 1 Interview questions.

## Data Availability

The data for this study (eg, interview transcripts) are not publicly available as per guidelines from the host institution’s research ethics board. Data access is restricted to help safeguard the privacy and confidentiality of the participants.
