# Supplementary material for: Health Professionals’ Views on the Use of Conversational Agents for Health Care: Qualitative Descriptive Study
Source: J Med Internet Res. 2024 Sep 25;26:e49387. doi: 10.2196/49387 (PMC11464950; doi:10.2196/49387)
Supplement: Multimedia Appendix 1 [file jmir_v26i1e49387_app1.docx]

**Interview Questions**

(1) Please describe your role as a health professional.

(2) Have you ever used a conversational agent in your personal life (for instance, a customer service chatbot or a voice-activated virtual assistant like Siri or Alexa)?

(3) Have you ever used a conversational agent that is used for health care specifically?

(4) Think about the use of conversational agents for health care.

(a) What benefits would these programs have for patients or health seekers?

(b) What drawbacks would these programs have for patients or health seekers?

(c) What benefits would these programs have for health professionals?

(d) What drawbacks would these programs have for health professionals?

(5) Think about conversational agents that are designed to simulate health professionals (for instance, they are given the title and appearance of a health professional).

(a) What advantages do you see (if any) to having conversational agents simulate health professionals?

(b) What concerns do you have (if any) about conversational agents that simulate health professionals?

(6) Should conversational agents be used for health care (in general)? Why or why not?

(7) Should conversational agents simulate health professionals? Why or why not?

(8) In your opinion, what would be the best way to integrate conversational agents into the health care system?

(9) Is there anything else you would like to share about the use of conversational agents for health care?

Sample probing questions/statements:

- What do you mean by that?
- Tell me more about that.
- How do you feel about that?
- Can you give me an example?
